# Carotid Intima–media Thickness is Lower after Sleeve Gastrectomy, with Concurrent Changes in Oxidized LDL and PAI-1

**DOI:** 10.1007/s11695-026-08538-z

**Published:** 2026-03-11

**Authors:** Mohamed Hany, Bart Torensma, Ramy E. Arida, Hala M. Demerdash, Mahmoud Ibrahim, Ahmed Shaaban, Zeyad Mohamed Hany, Mohamed N. Roushdy

**Affiliations:** 1 Department of Surgery, Medical Research Institute, Alexandria, Egypt; 2 Madina Women’s Hospital, Alexandria, Egypt; 3 Clinical Epidemiology, Erasmus MC, Rotterdam, Netherlands; 4 Alexandria University Hospitals, Alexandria, Egypt; 5 Department of Clinical Pathology, Alexandria University Hospitals, Alexandria, Egypt; 6 Department of Radiology, Alexandria University Student Hospital, Alexandria, Egypt; 7 Student, Alexandria University, Alexandria, Egypt; 8 Lecturer, Medical Military Academy, Alexandria Armed Forces Hospital, Alexandria, Egypt; 9 Consultant of Medical Microbiology and Clinical Immunology, Alexandria Armed Forces Hospital, Alexandria, Egypt

**Keywords:** Leptin, Plasminogen activator inhibitor, Oxidized LDL, CIMT, Inflammation, Fibrinolysis, Cardiovascular risk

## Abstract

**Background:**

Obesity is a chronic condition characterized by low-grade systemic inflammation, oxidative stress, and impaired fibrinolysis, all of which contribute to elevated cardiovascular risk. This study aimed to investigate the association between carotid intima-media thickness (CIMT), oxidized Low-Density Lipoprotein (ox-LDL), plasminogen activator inhibitor-1 (PAI-1), and inflammatory biomarkers in patients with obesity undergoing sleeve gastrectomy (SG), a widely performed metabolic and bariatric surgery (MBS) procedure.

**Methods:**

This prospective study included 93 patients with obesity who underwent SG. CIMT, body mass index (BMI), and waist-hip ratio were measured preoperatively and one year postoperatively. Concurrently, serum levels of ox-LDL, PAI-1, lipid profile, HOMA-IR, leptin, and high-sensitivity C-reactive protein (hs-CRP) were assessed.

**Results:**

One year after surgery, significant reductions were observed in CIMT, anthropometric parameters, inflammatory biomarkers (leptin, hs-CRP), insulin resistance, ox-LDL, and PAI-1 levels, along with improvement in lipid profile. PAI-1 was positively correlated with ox-LDL (MD: 5.94, 95% CI: 4.48–7.39; *p* < 0.001), and ox-LDL was a predictor of PAI-1 levels (MD: 0.05, 95% CI: 0.04–0.06; *p* < 0.001). In ROC analysis for predicting CIMT ≥ 1 mm, ox-LDL showed acceptable discriminative ability (AUC: 0.73; sensitivity: 82.1%, specificity: 64.8%), while PAI-1 demonstrated limited performance (AUC: 0.64; sensitivity: 48.7%, specificity: 79.6%).

**Conclusion:**

Weight loss following SG was associated with improvement in inflammatory, oxidative, and fibrinolytic biomarkers. Ox-LDL was more strongly linked to CIMT than PAI-1, which showed limited predictive value. Further studies are needed to evaluate the relationship between CIMT and other fibrinolysis biomarkers such as thrombin-activatable fibrinolysis inhibitor (TAFI) and D-dimer in the context of MBS.

## Key Points

1. PAI-1 and ox-LDL levels significantly decreased one year after SG, alongside improvements in inflammatory and insulin resistance markers.
2. A strong bidirectional association was found between ox-LDL and PAI-1, suggesting a mechanistic link between oxidative stress and fibrinolysis in obesity.
3. Ox-LDL demonstrated acceptable predictive performance for CIMT ≥ 1 mm (AUC: 0.73, sensitivity: 82.1%, specificity: 64.8%), indicating its potential as a vascular risk marker.
4. PAI-1 showed poor discriminative ability for subclinical atherosclerosis (AUC: 0.64), limiting its utility in cardiovascular risk prediction post-MBS.
5. These results support the integration of ox-LDL, rather than PAI-1, into future biomarker panels aimed at evaluating vascular improvement following MBS.

## Introduction

Obesity is a chronic disease associated with a spectrum of metabolic complications such as type 2 diabetes mellitus, dyslipidemia, and nonalcoholic fatty liver disease [1, 2]. It also confers a markedly elevated risk for cardiovascular diseases (CVDs), including hypertension, arterial and venous thrombosis, atherosclerosis, and stroke [3, 4]. Obesity-related risk factors are interconnected and often lead to multi-organ dysfunction. Managing obesity requires a long-term, multimodal approach, considering personal therapeutic goals and lifestyle changes [5]. Additionally, metabolic surgery can facilitate rapid weight loss and improve health outcomes [6].

The underlying pathophysiological mechanisms involve chronic low-grade inflammation, oxidative stress, and impaired fibrinolysis. Plasminogen activator inhibitor-1 (PAI-1), a member of the serine protease inhibitor (serpin) family [7], is the primary inhibitor of the fibrinolytic system, regulating the activity of tissue plasminogen activator (t-PA) and urokinase-type plasminogen activator (u-PA) [8, 9]. These enzymes catalyze the conversion of plasminogen to plasmin, responsible for the degradation of fibrin clots [9, 10]. Beyond its antifibrinolytic properties, PAI-1 plays a multifaceted role in inflammation, extracellular matrix turnover, tissue remodeling, angiogenesis, and cell migration, partially through modulation of matrix metalloproteinases (MMPs) [10–12].

PAI-1 is secreted by a range of cells, including endothelial and smooth muscle cells, hepatocytes, fibroblasts, megakaryocytes, cardiac myocytes, adipocytes, and macrophages [12, 13]. Following synthesis, it circulates in two major pools: free in plasma and stored within platelets [13, 14]. The circulating concentration of PAI-1 typically exceeds t-PA levels in a 4:1 ratio, and it is primarily cleared by hepatic uptake [12, 14].

In individuals with obesity, visceral adipose tissue becomes a major source of PAI-1 due to increased secretion by stromal cells and infiltrating macrophages, particularly in response to pro-inflammatory stimuli such as Tumor Necrosis Factor-alpha (TNF-α), Interleukin-6 (IL-6), and leptin [12, 13]. PAI-1 expression is also influenced by adipose tissue mass, insulin resistance, circulating insulin, and glucocorticoids [13–15]. This elevated expression contributes to a hypofibrinolytic, prothrombotic state that may promote atherosclerosis by limiting fibrin degradation and modulating cell adhesion and migration in the vascular wall [7, 13].

Several studies have suggested a potential association between PAI-1 and lipid metabolism, particularly very low-density lipoprotein (VLDL), which appears to enhance PAI-1 transcription via a response element in its promoter region [16, 17]. While links between PAI-1 and low-density lipoprotein (LDL) have also been proposed, evidence remains inconsistent and poorly defined.

Low-density lipoprotein (LDL) is the primary carrier of cholesterol to peripheral tissues and a major contributor to atherogenesis. Oxidized LDL (ox-LDL), formed through oxidative modification of LDL particles, is highly atherogenic and capable of activating inflammatory pathways and endothelial dysfunction [18, 19]. Circulating ox-LDL contains peroxidized lipids and reactive aldehydes that further amplify vascular injury and may influence PAI-1 expression and thrombotic risk.

In contrast, high-density lipoprotein cholesterol (HDL-C) exerts protective effects against atherothrombosis by promoting reverse cholesterol transport, suppressing endothelial inflammation, and inhibiting oxidative stress [20]. Higher HDL-C levels are inversely associated with PAI-1 expression, possibly through modulation of endothelial function and inflammatory signaling [21]. The anti-inflammatory and antioxidant properties of HDL-C may attenuate PAI-1 induction in vascular tissues, thereby contributing to improved fibrinolytic balance and reduced cardiovascular risk [22].

Subclinical atherosclerosis is commonly observed in patients with obesity, even in the absence of clinically manifest cardiovascular disease [23]. Carotid intima-media thickness (CIMT), assessed via ultrasound, is a validated surrogate marker of early vascular aging, endothelial dysfunction, and low-grade systemic inflammation [24]. As a noninvasive, reproducible tool, CIMT provides a quantitative measure of early atherosclerotic changes and has been shown to predict future cardiovascular events following metabolic and bariatric surgery (MBS) [24–27]. These data suggest that early vascular alterations can be captured by integrating imaging techniques and circulating molecular biomarkers reflecting systemic and metabolic derangements.

Among these biomarkers, high-sensitivity C-reactive protein (hs-CRP) plays a central role as an established indicator of vascular inflammation and cardiometabolic risk [28]. Elevated hs-CRP levels are associated with increased endothelial dysfunction, insulin resistance, and progression of atherosclerosis [29–31]. Recent findings by Hany et al. demonstrated that postoperative reductions in hs-CRP levels following SG were significantly correlated with improvements in cardiovascular risk markers, including favorable shifts in lipid profiles (LDL-C/HDL-C) and metabolic ratios [32]. Notably, these changes were observed even in patients with modest BMI reductions, underscoring the relevance of inflammation resolution as a key mechanism of cardiovascular benefit [32]. These findings reinforce the utility of hs-CRP as a dynamic, weight-independent marker of vascular risk recovery after MBS [29, 32]. Sleeve gastrectomy (SG), currently the most frequently performed MBS procedure worldwide [33–35], not only induces weight loss but also improves inflammation, insulin sensitivity, and oxidative stress parameters, which may impact fibrinolytic balance and vascular risk [36–38].

We hypothesized that increased oxidative stress resulting from elevated ox-LDL and inflammatory factors would lead to elevated PAI-1 production in adipose and endothelial cells, thereby impairing fibrinolysis and increasing cardiovascular risk in obesity, as indicated by increased CIMT. This study aimed to explore the relationship between CIMT, ox-LDL, and PAI-1 as cardiovascular risk markers in obese patients undergoing sleeve gastrectomy. Additionally, we examined the associations between these biomarkers and anthropometric indices, insulin resistance (HOMA-IR), lipid profiles, and inflammatory markers like leptin and hs-CRP in the context of MBS.

## Methods

### Study Design and Ethical Approval

This was a single-center, prospective cohort study conducted at [BLINDED], between January 2024 and March 2024, with registration number IORG0008812 E/C. S/N. R12/2023. Eligible participants were adults with obesity scheduled to undergo SG. The study was conducted following the Declaration of Helsinki and received approval from the institutional ethics committee. All participants provided written informed consent before enrollment and data collection.

### Eligibility Criteria

Participants were recruited from patients referred to SG during the study period. Inclusion criteria comprised adults aged over 18 years with a body mass index (BMI) > 35 kg/m², or BMI > 30 kg/m² with at least one obesity-associated disease such as type 2 diabetes mellitus, dyslipidemia, or hypertension, following the AMSBS/IFSO 2022 guidelines [39].

Exclusion criteria included a history of clinically manifest atherosclerotic cardiovascular disease, known coagulopathies, or any current malignancy. These criteria were applied to minimize confounding and ensure a homogenous population for assessing subclinical cardiovascular risk.

### Data Collection and Follow-up

Participants underwent structured assessments at two time points: preoperatively, within one week before SG, and at 12 months postoperatively. Data collection included anthropometric measurements, laboratory investigations, and carotid intima-media thickness (CIMT) evaluation.

Anthropometric parameters, including body weight in kg, height in m, BMI (kg/m²), waist circumference (WC), and hip circumference (HC), were measured. Waist-to-hip ratio (WHR) was calculated, with thresholds defined according to World Health Organization (WHO) criteria for metabolic risk [40] with WC ≥ 94 cm (men) or ≥ 80 cm (women) indicating increased risk; ≥102 cm (men) or ≥ 88 cm (women) indicating substantially increased risk; WHR ≥ 0.90 (men) or ≥ 0.85 (women) indicating elevated cardiovascular risk [40]. Waist-to-height ratio (WHtR) was also calculated as WC (cm)/height (cm) [41].

Participants were followed clinically every three months during the 12 months to ensure adherence, monitor postoperative course, and collect interval medical history. Concomitant cardiometabolic medication exposure during follow-up (e.g., statins, antiplatelet agents including aspirin, antihypertensives, and glucose-lowering therapies) was not systematically captured. Outcome assessments were performed at the 12-month visit.

### Laboratory Investigations

Fasting venous blood samples were collected in the early morning, one week before surgery, and at 12 months postoperatively. Samples were processed immediately, with aliquots stored at − 80 °C until analysis. All assays were performed in a single laboratory using standardized protocols to ensure consistency.

Routine biochemistry included fasting plasma glucose and lipid profile (total cholesterol, triglycerides, HDL-cholesterol) measured using the Hitachi 7180 Biochemistry Automatic Analyzer (Hitachi, Japan). LDL-cholesterol was calculated using the Friedewald equation [42]: LDL = Total Cholesterol − HDL − (Triglycerides/5), provided triglycerides < 400 mg/dL.

Fasting insulin concentrations were quantified using a commercially available ELISA kit (DRG International, Inc., EIA-2935, Springfield, NJ, USA). Insulin resistance was estimated using the Homeostasis Model Assessment of Insulin Resistance (HOMA-IR): HOMA-IR = [Fasting Insulin (µIU/mL) × Fasting Glucose (mmol/L)]/22.5.

Serum leptin levels were measured using an ELISA kit (Cloud-Clone Corp; Cat. No. E-00916hu, TX 77494, USA), and high-sensitivity C-reactive protein (hs-CRP) was determined by nephelometry (Behring Diagnostics, Marburg, Germany).

Serum PAI-1 concentrations were assessed using a sandwich ELISA (Chongqing Biospes Co., Ltd; Cat. No. BZEK1701), while oxidized LDL (ox-LDL) was measured using a separate ELISA kit from the same manufacturer (Cat. No. BZEK2460). All samples were run in duplicate, and the average value was used for analysis.

### Carotid Intima-media Thickness (CIMT) Assessment

High-resolution B-mode ultrasonography was performed using a Philips HD 12 Digital Ultrasound system by a single radiologist blinded to the study objectives. Participants were examined in the supine position with the neck extended and slightly rotated to optimize visualization of the carotid arteries.

Bilateral CIMT measurements were obtained from the common carotid artery (CCA) at three standardized locations: (1) proximal segment (1 cm proximal to the carotid bifurcation), (2) middle segment (4 cm from the bifurcation), and (3) distal segment (first 1 cm of the carotid bulb). Measurements were taken from anterior, posterior, and lateral views using longitudinal imaging, ensuring the far wall was parallel to the ultrasound beam and the lumen diameter was maximized.

Mean CIMT values were calculated from the three sites on both sides and expressed in millimeters. The Doppler mode was used to confirm laminar flow. Assessments were conducted at baseline (preoperative) and 12 months postoperatively.

A CIMT value ≥ 1.0 mm was considered indicative of increased absolute cardiovascular risk, consistent with the American Society of Echocardiography (ASE) criteria [43].

### Surgical Procedure

All SG procedures were performed by a single experienced surgical team using a standardized laparoscopic approach. Pneumoperitoneum was established using optical trocar entry, followed by placement of five ports: three 12-mm ports (camera and bilateral working ports) and two 5-mm ports (for liver retraction and assistance).

The greater curvature of the stomach was mobilized beginning approximately 3–5 cm from the pylorus and extending to the angle of His. The greater omentum was dissected using the EnSeal device (Ethicon Endo-Surgery, Cincinnati, OH, USA), and any posterior gastric adhesions were released. Belsey’s pad of fat was excised when present to facilitate exposure.

Gastric calibration was achieved using a 36-Fr bougie placed along the lesser curvature. Gastric transection was performed using the Echelon Flex Endopath 60-mm linear stapler (Ethicon Endo-Surgery, Cincinnati, OH, USA), employing green, gold, and blue cartridges according to tissue thickness.

The staple line was fully invaginated with a running seromuscular suture using unidirectional absorbable 3/0 V-Loc™ 180 sutures (Covidien, Mansfield, MA, USA). In cases where a hiatal hernia was identified intraoperatively, posterior cruroplasty was performed prior to gastric transection.

### Sample Size Calculation

Sample size estimation was performed a priori to ensure sufficient power for Receiver Operating Characteristic (ROC) curve analysis. Using the pROC package in R, the calculation targeted an expected area under the curve (AUC) of 0.70 to assess the diagnostic accuracy of serum biomarkers in predicting elevated carotid intima-media thickness (CIMT). With a significance level (α) of 0.05 and statistical power of 80%, the minimum required sample was 31 participants with CIMT ≥ 1 mm and 31 with CIMT < 1 mm. To account for dropouts and variability, 93 participants were ultimately enrolled, ensuring robust statistical inference.

### Statistical Analysis

All statistical analyses were conducted using R software (version 4.4.2) and MedCalc (version 12.4.0.0) for ROC curve evaluation. Descriptive statistics were used to summarize baseline characteristics, including means, standard deviations, ranges, and proportions.

Longitudinal changes in anthropometric, biochemical, and vascular parameters from baseline to 12 months were analyzed using Generalized Estimating Equations (GEE), accounting for within-subject correlation over time. GEE models were also applied to examine associations between biomarkers and continuous outcomes, with results reported as mean differences (MD) and corresponding 95% confidence intervals (CI), adjusted for time, age, and gender.

To evaluate the ability of biomarkers to predict elevated CIMT (≥ 1 mm), logistic GEE models were used to estimate adjusted odds ratios (OR) and 95% CI. ROC curve analyses were performed to determine the discriminative performance of PAI-1 and ox-LDL, with AUC values and 95% CI calculated. Youden’s index was used to determine the optimal cutoff points maximizing sensitivity and specificity.

Comparisons between AUCs of biomarkers were statistically tested to assess superiority in predictive accuracy. All p-values were two-tailed, with statistical significance set at *p* < 0.05.

## Results

### Baseline Characteristics

Ninety-three patients (82.8% female) with obesity were enrolled. The mean age was 39.6 ± 9.4 years, and the baseline BMI was 46.8 ± 7.3 kg/m². Elevated cardiovascular risk was prevalent at baseline, with a mean CIMT of 1.0 ± 0.2 mm, and 41.9% (39 patients) had a CIMT ≥ 1 mm. Mean WHR was 0.92 ± 0.08, and 85% exceeded the WHO thresholds for central adiposity. Inflammatory markers were also elevated: mean serum leptin was 39.3 ± 6.3 ng/mL, and hs-CRP was 6.7 ± 2.3 mg/L (Table 1).

**Table 1:** Baseline characteristics of the participants (*N* = 93)

Variable	Value
Age (years)	39.6 ± 9.4
Sex	
Female	77 (82.8)
Male	16 (17.2)
Anthropometrics	
Weight (kg)	126.9 ± 23.1
BMI (kg/m^2^)	46.8 ± 7.3
Waist circumference (cm)	130.2 ± 13.1
Hip circumference (cm)	137.7 ± 11.6
Waist/hip ratio	0.95 ± 0.03
Associated medical illnesses	
Obstructive Sleep Apnea	62 (66.7)
Dyslipidemia	58 (62.4)
Insulin resistance	51 (54.8)
Osteoarthritis	46 (49.5)
Hypertension	43 (46.2)
COPD	24 (25.8)
Menstrual irregularities	14 (15.1)
GERD	10 (10.8)
Hypothyroidism	9 (9.7)
IBD	6 (6.5)
Congestive Heart failure	4 (4.3)
History of DVT	3 (3.2)
Rheumatoid disease	3 (3.2)
Gout	2 (2.2)
HCV positive	2 (2.2)
HBsAg positive	1 (1.1)
CIMT Sonar (mm)	1.0 ± 0.2
CIMT ≥ 1 mm	39 (41.9)

Cell values represent frequency (%) or mean ± standard deviation. *CIMT* Carotid Intima-Media Thickness, *BMI* Body Mass Index, *COPD* Chronic Obstructive Pulmonary Disease, *GERD* Gastroesophageal Reflux Disease, *IBD* Inflammatory Bowel Disease, *DVT* Deep Vein Thrombosis

### Postoperative Changes in One Year

Twelve months after SG, patients exhibited significant reductions in BMI (−16.4 kg/m²), WHR (− 0.10), and CIMT (0.05 mm, *p* < 0.001). Leptin and hs-CRP levels declined significantly, with improved insulin sensitivity (HOMA-IR reduction: −2.8, *p* < 0.001). Lipid profile showed favorable changes: LDL and triglycerides decreased; HDL increased (Table 2).

**Table 2:** Changes in anthropometrics and lab investigations at post-surgical year 1 estimated by GEE analyses

Variable	Pre-surgery (*n* = 93)	Year 1 Post-surgery (*n* = 93)	MD (95% CI)	*p*
Anthropometrics				
Weight (kg)	126.9 ± 23.1	83.0 ± 15.6	−44.0 (−49.6, −38.3)	< 0.001*
BMI (kg/m^2^)	46.8 ± 7.3	30.5 ± 4.8	−16.4 (−18.1, −14.6)	< 0.001*
Waist circumference (cm)	130.2 ± 13.1	100.0 ± 13.0	−30.1 (−33.9, −26.4)	< 0.001*
Hip circumference (cm)	137.7 ± 11.6	118.5 ± 11.6	−19.2 (−22.6, −15.9)	< 0.001*
WHtR ratio	0.95 ± 0.0	0.8 ± 0.0	−0.10 (−0.12, −0.09)	< 0.001*
CBC				
Hemoglobin (mg/dl)	12.4 ± 1.4	12.5 ± 1.5	0.2 (−0.2, 0.6)	0.412
Platelets (x10^9^/L)	297.5 ± 77.9	253.0 ± 62.6	−44.6 (−64.8, −24.4)	< 0.001*
WBCs (x10^9^/L)	14.6 ± 6.67	6.3 ± 1.9	−8.3 (−21.8, 5.2)	0.228
Lipid profile				
Total Cholesterol (mg/dl)	216.6 ± 21.9	184.7 ± 11.3	−31.9 (−36.9, −26.9)	< 0.001*
LDL-C (mg/dl)	138.0 ± 15.9	110.5 ± 7.6	−27.5 (−31.1, −24.0)	< 0.001*
HDL-C (mg/dl)	40.9 ± 3.5	51.2 ± 2.9	10.4 (9.4, 11.3)	< 0.001*
Triglycerides (mg/dl)	191.7 ± 61.6	113.2 ± 20.9	−78.6 (−91.7, −65.4)	< 0.001*
Inflammatory biomarkers				
Leptin (ng/ml)	39.3 ± 6.3	17.3 ± 4.0	−21.9 (−23.5, −20.4)	< 0.001*
hs-CRP (mg/L)	6.7 ± 2.3	2.5 ± 0.8	−4.2 (−4.7, −3.7)	< 0.001*
Insulin Resistance				
FBG (mg/dl)	123.0 ± 31.2	89.0 ± 12.3	−34.0 (−40.8, −27.2)	< 0.001*
Insulin (IU/ml)	13.2 ± 8.3	6.2 ± 2.9	−7.0 (−8.8, −5.2)	< 0.001*
HOMA IR	4.2 ± 3.3	1.4 ± 1.3	−2.8 (−3.5, −2.1)	< 0.001*
Fibrinolysis/oxidative stress Biomarkers				
PAI-1(pg/ml)	2317.1 ± 464.2	1023.3 ± 143.5	−1293.9 (−1392.1, −1195.7)	< 0.001*
Ox-LDL(µg/ml)	224.7 ± 26.4	117.3 ± 36.7	−107.5 (−116.6, −98.3)	< 0.001*
pre-CIMT Sonar (mm)	1.0 ± 0.2	0.6 ± 0.2	−0.4 (−0.4, −0.3)	< 0.001*
CIMT ≥ 1 mm (OR)	39 (41.9)	3 (3.2)	0.05 (0.01, 0.16)	< 0.001*

Cell values represent Mean ± standard deviation or frequency (%); *WH* *ratio* Waist/Hip ratio, *WHtR* waist circumference/height, *WBCs* White blood cells, *HOMA* *IR* Homeostatic Model Assessment of Insulin Resistance, *hs*-*CRP* high-sensitivity C-reactive protein, *FBG* Fasting blood glucose, *PAI*-*1* Plasminogen activator inhibitor-1, *ox*-*LDL* oxidized LDL, *CIMT* Carotid Intima-Media Thickness, *HDL* *C* High-Density Lipoprotein Cholesterol, *LDL* *C* High-Density Lipoprotein Cholesterol, *BMI* Body Mass Index, *MD* Mean difference (Year 1 – Baseline), *OR* Odds ratio (Year 1 vs. baseline), *CI* confidence interval. *Statistically significant (*p* < 0.05)

**Fig. 1:**
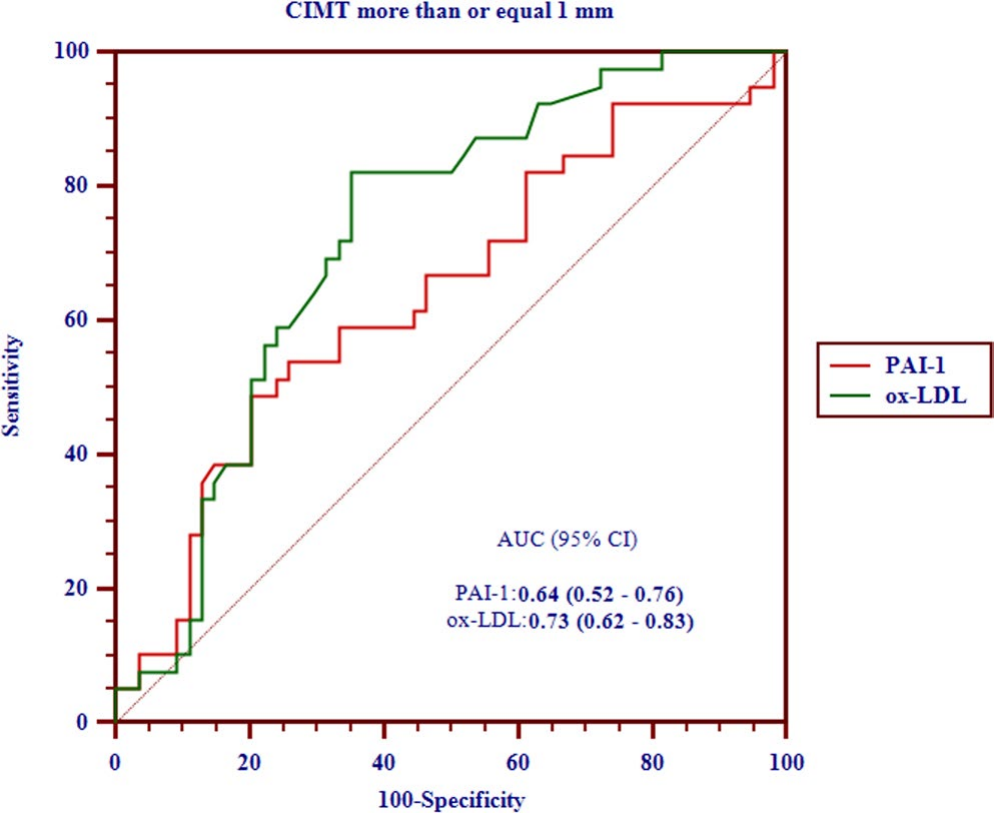
Receiver Operating Characteristic (ROC) curves comparing the diagnostic performance of PAI-1 and ox-LDL in predicting carotid intima-media thickness (CIMT) of ≥ 1 mm. This figure illustrates the sensitivity and specificity of each biomarker at various decision thresholds, highlighting ox-LDL’s superior diagnostic accuracy (AUC: 0.73, 95% CI: 0.62–0.83) compared to PAI-1, which demonstrates poor performance

Oxidative and fibrinolytic markers improved substantially. Ox-LDL levels decreased by 107.5 µg/ml (*p* < 0.001), and PAI-1 levels decreased by 1293.9 pg/ml (*p* < 0.001). These changes were accompanied by significant shifts in cardiovascular risk classification: the proportion of patients with CIMT ≥ 1 mm fell from 41.9% to 3.2% (Table 2) (Fig. 1).

### Predictors of CIMT and CIMT ≥ 1 mm

In multivariable GEE models, WHR was an independent predictor of CIMT (MD = 1.08, 95% CI: 0.37–1.79, *p* = 0.003), along with hs-CRP (MD = 0.03, 95% CI: 0.01–0.05, *p* < 0.001). Elevated WHR was also associated with increased odds of having CIMT ≥ 1 mm (adjusted OR [AOR]: 2.7 × 10⁷, 95% CI: 10.29–7.2 × 10¹³, *p* = 0.023). Serum leptin levels were also positively associated with CIMT (MD = 0.01, 95% CI: 0.01–0.02, *p* < 0.001) (Table 3).

**Table 3:** Factors associated with CMIT and odds of having CMIT ≥ 1 mm estimated by generalized estimating equations

Predictor	CMIT (mm)		CMIT ≥ 1 mm	
	MD (95% CI)	*p*	AOR (95% CI)	*p*
Fibrinolysis/oxidative stress Biomarkers				
PAI-1 (pg/ml)	0.0001 (0.0001, 0.0002)	< 0.001*	1.00 (1.00, 1.00)	0.045*
ox-LDL (µg/ml)	0.002 (0.002, 0.003)	< 0.001*	1.02 (1.01, 1.04)	0.011*
Anthropometrics				
BMI (kg/m^2^)	0.01 (0.01, 0.02)	< 0.001*	1.12 (1.04, 1.20)	0.001*
Waist/hip ratio	1.08 (0.37, 1.79)	0.003*	2.7 × 10^07^ (10.29, 7.2 × 10^13^)	0.023*
Inflammatory biomarkers				
Leptin (ng/ml)	0.01 (0.01, 0.02)	< 0.001*	1.07 (1.00, 1.14)	0.055*
hs-CRP (mg/L)	0.03 (0.01, 0.05)	< 0.001*	1.49 (1.19, 1.87)	0.001*
Insulin resistance				
HOMA IR	0.02 (0.00, 0.03)	0.007*	1.17 (1.03, 1.33)	0.018*
Lipid profile				
Cholesterol (mg/dl)	0.002 (0.001, 0.004)	0.003*	1.02 (1.00, 1.04)	0.034*
LDL C (mg/dl)	0.002 (0.000, 0.004)	0.084	1.02 (0.99, 1.05)	0.145
HDL C (mg/dl)	0.00 (−0.01, 0.01)	0.519	1.00 (0.89, 1.13)	0.959
Triglycerides (mg/dl)	0.001 (0.000, 0.001)	0.073	1.00 (1.00, 1.01)	0.316

*MD* Mean difference in CMIT adjusted for time, age, and sex, *AOR* odds ratio of having CMIT ≥ 1 mm adjusted for time, age, and sex. *Statistically significant (*p* < 0.05). *HOMA* *IR* Homeostatic Model Assessment of Insulin Resistance, *hs*-*CRP* high-sensitivity C-reactive protein, *PAI*-*1* Plasminogen activator inhibitor-1, *ox*-*LDL* oxidized LDL, *CIMT* Carotid Intima-Media Thickness, *HDL* *C* High-Density Lipoprotein Cholesterol, *LDL* *C* High-Density Lipoprotein Cholesterol, *BMI* Body Mass Index

### Associations of PAI-1 and Ox-LDL with Cardiometabolic Parameters

Both ox-LDL and PAI-1 were significantly associated with markers of inflammation and dyslipidemia. Ox-LDL was positively associated with total cholesterol (MD = 0.58, 95% CI: 0.36–0.80, *p* < 0.001), LDL-C (MD = 0.58, 95% CI: 0.29–0.88, *p* < 0.001), triglycerides (MD = 0.15, 95% CI: 0.01–0.28, *p* = 0.035), hs-CRP (MD = 6.13, 95% CI: 4.24–8.03, *p* < 0.001), and HOMA-IR (MD = 4.04, 95% CI: 2.34–5.75, *p* < 0.001) (Table 4).

**Table 4:** Factors associated with PAI-1 and ox-LDL estimated by generalized estimating equations

Predictor	PAI-1		ox-LDL	
	MD (95% CI)	*p*	MD (95% CI)	*p*
Anthropometrics				
BMI (kg/m^2^)	39.43 (32.60, 46.25)	< 0.001*	4.48 (4.12, 4.84)	< 0.001*
Waist circumference (cm)	10.23 (6.27, 14.19)	< 0.001*	1.59 (1.35, 1.83)	< 0.001*
Hip circumference (cm)	10.99 (6.50, 15.48)	< 0.001*	1.66 (1.38, 1.93)	< 0.001*
Waist/hip ratio	1963.70 (910.93, 3016.46)	< 0.001*	338.54 (214.57, 462.50)	< 0.001*
Lipid profile				
Cholesterol (mg/dl)	5.09 (2.22, 7.95)	< 0.001*	0.58 (0.36, 0.80)	< 0.001*
LDL C (mg/dl)	7.38 (3.25, 11.50)	< 0.001*	0.58 (0.29, 0.88)	< 0.001*
HDL C (mg/dl)	7.11 (−10.18, 24.40)	0.420	1.00 (−0.51, 2.51)	0.196
Triglycerides (mg/dl)	0.49 (−1.04, 2.03)	0.528	0.15 (0.01, 0.28)	0.035*
Inflammatory biomarkers				
Leptin (ng/ml)	27.70 (17.84, 37.56)	< 0.001*	2.87 (2.12, 3.62)	< 0.001*
hs-CRP (mg/L)	73.88 (42.33, 105.44)	< 0.001*	6.13 (4.24, 8.03)	< 0.001*
Insulin resistance				
HOMA IR	53.99 (30.01, 77.98)	< 0.001*	4.04 (2.34, 5.75)	< 0.001*
Fibrinolysis/oxidative stress Biomarkers				
ox-LDL (µg/ml)	5.94 (4.48, 7.39)	< 0.001*		
PAI-1 (pg/ml)			0.05 (0.04, 0.06)	< 0.001*

*MD* Mean difference adjusted for time, age, and sex. *CI* confidence interval. *Statistically significant (*p* < 0.05). *HOMA* *IR* Homeostatic Model Assessment of Insulin Resistance, *hs*-*CRP* high-sensitivity C-reactive protein, *PAI*-*1* Plasminogen activator inhibitor-1, *ox*-*LDL* oxidized LDL, *HDL* *C* High-Density Lipoprotein Cholesterol, *LDL* *C,* High-Density Lipoprotein Cholesterol, *BMI* Body Mass Index

Similarly, PAI-1 levels were positively associated with total cholesterol (MD = 5.09, 95% CI: 2.22–7.95, *p* < 0.001), LDL-C (MD = 7.38, 95% CI: 3.25–11.50, *p* < 0.001), hs-CRP (MD = 73.88, 95% CI: 42.33–105.44, *p* < 0.001), and HOMA-IR (MD = 53.99, 95% CI: 30.01–77.98, *p* < 0.001); triglycerides were not significantly associated with PAI-1 (MD = 0.49, 95% CI: −1.04 to 2.03, *p* = 0.528) (Table 4).

Bidirectional modeling confirmed a mutual association: higher PAI-1 predicted increased ox-LDL (MD = 5.94, 95% CI: 4.48–7.39, *p* < 0.001), and ox-LDL predicted higher PAI-1 levels (MD = 0.05, 95% CI: 0.04–0.06, *p* < 0.001) (Table 4).

### Diagnostic Accuracy of PAI-1 and Ox-LDL for CIMT ≥ 1 mm

Receiver operating characteristic (ROC) analyses for baseline CIMT ≥ 1 mm revealed that ox-LDL had acceptable performance (AUC: 0.73, 95% CI: 0.62–0.83), with a sensitivity of 82.1%, specificity of 64.8%, and an optimal threshold at > 219.5 µg/mL. In contrast, PAI-1 showed lower discriminative ability (AUC: 0.64, 95% CI: 0.52–0.76), with sensitivity of 48.7% and specificity of 79.6% at the > 2433.5 pg/mL cutoff. Ox-LDL had a higher negative predictive value (NPV: 83.3%) compared with PAI-1 (NPV: 68.3%), while positive predictive values were comparable (PPV: 62.7% for ox-LDL vs. 63.3% for PAI-1) (Table 5). The AUC difference (ox-LDL minus PAI-1) was 0.09 (95% CI: 0.00–0.17; *p* = 0.050).

**Table 5:** Diagnostic test accuracy indices of ox-LDL and PAI-1 in predicting CIMT ≥ 1 mm using the sample lab results at year 1 and baseline, respectively

CIMT threshold (mm)	Indices	ox-LDL	PAI-1
≥ 1 mm (baseline) Positive cases = 39 Negative cases = 54	AUC	0.73 (0.62–0.83)	0.64 (0.52–0.76)
	Best threshold	> 219.5	> 2433.5
	Sensitivity	82.1% (66.5–92.5%)	48.7% (32.4–65.2%)
	Specificity	64.8% (50.6–77.3%)	79.6% (66.5–89.4%)
	PPV	62.7% (48.1–75.9%)	63.3% (43.9–80.1%)
	NPV	83.3% (68.4–93.1%)	68.3% (55.2–79.5%)
	+ LR	2.3 (1.6–3.4)	2.4 (1.3–4.4)
	- LR	0.3 (0.1–0.6)	0.6 (0.5–0.9)
	∆ AUC _ox−LDL – PAI−1_ (95% CI)	0.09 (0.00 to 0.17), *p* = 0.050	

*CIMT* Carotid Intima-Media Thickness, *AUC* area under the ROC curve, *PPV* positive predictive value, *NPV* negative predictive value, + *LR* positive likelihood ratio, - *LR* negative likelihood ratio, ∆ *AUC* difference in AUC

## Discussion

Numerous studies show a strong link between obesity and metabolic disturbances like dyslipidemia, insulin resistance, oxidative stress, inflammation, and impaired fibrinolysis, all of which contribute to cardiovascular diseases [44, 45].

Obesity-related vascular remodeling involves arterial stiffness and increased media thickness, affecting both large arteries like the aorta and smaller vessels such as the coronary arteries [44, 46]. This remodeling is driven in part by structural and functional changes in perivascular adipose tissue (PVAT), including increased PVAT mass, reduced vasodilatory signaling, enhanced vasoconstrictor activity, and increased oxidative stress [47, 48]. In obesity, PVAT adopts a pro-inflammatory secretory phenotype with dysregulated production of adipokines and growth factors (e.g., TNF-α and IL-6) together with other adipose-derived mediators such as PAI-1, resistin, visfatin, and altered nitric oxide synthase (NOS)/NO-related pathways; collectively, these changes can impair PVAT’s vasorelaxant function and promote endothelial dysfunction, vascular remodeling, antifibrotic and antiproliferative effects [49, 50]. Importantly, given the association-based nature of our analyses and the limited biomarker panel assessed, other unmeasured inflammatory mediators/adipokines (e.g., serum amyloid A, adiponectin-related pathways, complement factor H, and aquaporins) may also contribute to the relationships observed between inflammation, CIMT, ox-LDL, and PAI-1 [51–54].

Our study aimed to evaluate the influence of weight loss following SG on biomarkers of fibrinolysis and oxidative stress as they relate to cardiovascular risk. One year post-SG, significant reductions were observed in all anthropometric measurements, including BMI and WHR. Additionally, improvements in lipid profiles, HOMA-IR, and inflammatory markers, such as serum leptin and hs-CRP, were noted. This is consistent with the observations of Hany et al. [32], who reported that even modest reductions in BMI after SG were significantly associated with reductions in hs-CRP and MHR, along with improvements in the HDL-C/apo A-1 ratio [32]. These markers collectively underscore the role of inflammation and lipid remodeling in mediating cardiovascular risk reduction [32].

In terms of PAI-1, a key inhibitor of fibrinolysis, a substantial decline postoperatively was noted. Elevated preoperative levels of PAI-1 signal impaired fibrinolytic capacity and an increased thrombotic risk [12], with expression levels in visceral adipose tissue strongly correlating with central adiposity metrics [55] such as BMI (*p*<0.001) and waist-to-hip ratio (*p*<0.001). Our findings align with earlier studies linking PAI-1 to metabolic syndrome [55–57]. These studies suggest that obesity-related insulin resistance, heightened serum insulin, and elevated free fatty acid levels may reduce mRNA degradation of PAI-1, consequently stimulating its expression [55, 56].

Moreover, our study identified a significant correlation between PAI-1 and HOMA-IR (*p*<0.001). Insulin resistance is often accompanied by increased levels of very low-density lipoprotein (VLDL), which results in impaired clearance and induces PAI-1 gene transcription through MAPK pathway activation [57]. Serum PAI-1 levels also demonstrated a strong association with inflammatory markers, leptin (*p*<0.001) and hs-CRP (*p*<0.001). This association may stem from a chronic inflammatory state induced by adipocyte-derived cytokines, such as tumor necrosis factor-alpha (TNF-α) and interleukin-6 (IL-6), which in turn stimulate PAI-1 expression [56].

Eichinger et al. [58] emphasize the relationship between excess body weight and increased secretion of pro-inflammatory factors from adipose tissue, contributing to a hypercoagulable state and low-grade systemic inflammation, along with corresponding disruptions in the coagulation pathway, including impaired fibrinolysis [58]. This suggests that metabolic factors such as insulin resistance, BMI, and dyslipidemia may facilitate elevated PAI-1 expression [56].

Our lipid profile results indicated a significant association of total cholesterol and LDL cholesterol with PAI-1 levels, while HDL cholesterol and triglycerides did not exhibit significant correlations with PAI-1. This reinforces the notion that PAI-1’s relationship with LDL cholesterol, known to be associated with heightened cardiovascular risk, is particularly relevant in the context of obesity [59, 60]. Conversely, our study did not find an association between PAI-1 and HDL cholesterol, which is typically considered athero-protective [61].

Moreover, similar findings were reported by Somodi et al. [57], who found no significant correlation between PAI-1 and small HDL subfractions, specifically HDL3, using gel electrophoresis for lipoprotein subfraction analysis [57]. Notably, adipocytes from patients with obesity produce approximately twice as much PAI-1 compared to their lean counterparts [59].

In addition, the results demonstrated a significant reduction in ox-LDL levels (*p* < 0.001) one year post-surgery. Elevated preoperative ox-LDL, a biomarker indicative of lipoprotein-associated oxidative stress, is believed to activate circulating monocytes [62]. These monocytes adhere to the endothelium and migrate into the intima of the vascular wall, subsequently contributing to atherosclerotic plaque development [62]. Moreover, increased oxidative stress facilitates the conversion of LDL to more atherogenic ox-LDL, which serves as a potent activator of oxidative stress and inflammatory processes associated with atherosclerosis [63].

The findings of this study indicate that both BMI and WHR significantly correlated with levels of ox-LDL, suggesting that obesity, particularly central adiposity and insulin resistance, are major contributors to oxidative stress [63, 64]. Additionally, hypercholesterolemia and elevated LDL cholesterol were strongly associated with ox-LDL levels. Consistent with the findings of Xu et al. [65], which reported that statin therapy can reduce both LDL-cholesterol and ox-LDL levels [65]. Moreover, Several studies have also noted that insulin resistance and chronic hyperglycemia can induce oxidative stress, further modifying LDL cholesterol into proatherogenic ox-LDL [64].

Furthermore, our results highlight that weight reduction one year following SG corresponded with a significant decrease in anthropometric adiposity indices and inflammatory biomarkers, specifically leptin and hs-CRP, which were associated with reductions in ox-LDL levels. Given that chronic inflammation, oxidative stress, and resultant endothelial dysfunction are prevalent in obesity, the implications of these findings are profound [66, 67].

Circulating leptin concentrations are involved in the association between percentage body fat and cardiovascular risk factors, supporting its relevance as an adiposity-linked mediator of cardiometabolic risk [68]. Additionally, PAI-1 levels were linked to increased ox-LDL concentrations (MD: 5.94, *p* < 0.001), and reciprocally, ox-LDL affected PAI-1 levels (MD: 0.05, *p* < 0.001). Notably, lysophosphatidylcholine (LPC), a principal lipid component of ox-LDL, was shown to significantly stimulate aberrant expression of PAI-1 in adipocytes, potentially contributing to metabolic dysregulation in obesity [69].

In terms of CIMT, which serves as an independent marker for atherosclerotic disease, our results confirmed a positive correlation with PAI-1 levels, consistent with previous studies [70, 71]. Similarly, ox-LDL was associated with an increase in CIMT, aligning with the observations made by Dogan et al. [72]. Our findings further indicated a strong relationship between CIMT and waist-hip ratio, as well as significant associations between inflammatory biomarkers leptin and hs-CRP and changes in CIMT, suggesting resolution of low-grade inflammation following weight loss one year after SG [73].

ROC curve analysis for predicting CIMT thresholds of ≥ 1 mm revealed that ox-LDL demonstrated superior performance, reflected in an area under the curve (AUC) of 0.73, with a sensitivity of 82.1% and specificity of 64.8%, relative to PAI-1, which presented an AUC of 0.64, sensitivity of 48.7%, and a higher specificity of 79.6%.

Thus, elevated serum ox-LDL levels signify increased oxidative stress, which ultimately leads to vascular endothelial injury [16]. Furthermore, ox-LDL serves as a prominent ligand for macrophage scavenger receptors such as CD36 and SR-BI, enabling its infiltration into macrophages, where it is transformed into foam cells [44, 45]. Consequently, ox-LDL is implicated in the accumulation of lipids within macrophages, resulting in lipid-laden foam cells that contribute to the formation of atherosclerotic plaques [74]. These plaques can become lipid-overloaded and rupture, exposing subendothelial tissue and prompting platelet activation and aggregation [75]. This process initiates coagulation activation, leading to thrombosis and impaired fibrinolysis, which, in turn, contributes to atherosclerotic narrowing of the arteries [75–77]. Thus, the interplay of dyslipidemia, oxidative stress, and impaired fibrinolysis is recognized as a significant factor in increasing cardiovascular risk in obesity, with further improvements associated with weight loss.

Lastly, it is worth mentioning that MBS is associated with significant improvement of anthropometric and metabolic parameters. Together with concomitant resolution or improvement of obesity-related cardiovascular risk as a result of amelioration of insulin resistance, enhancement of lipid clearance, improved lipoprotein metabolism, and reduced oxidative stress, with subsequent reduction of inflammatory signals [32, 44, 45]. In this context, the present study offers a novel perspective on the interaction between fibrinolysis and oxidative stress pathways with respect to SG, with robust analysis of PAI-1 and ox-LDL as surrogate markers of vascular remodeling. The prospective design, detailed biomarker profiling, and one-year follow-up strengthen the internal validity and temporal inferences. The use of CIMT as a non-invasive surrogate of vascular risk adds clinical relevance and translational value.

Several limitations should be noted. This single-center study has a modest sample size, which may limit generalizability. We did not assess total or regional adiposity, particularly visceral fat, which is closely linked to inflammation and endothelial dysfunction; future studies should incorporate standardized measures of body composition and fat distribution. Additionally, the absence of adults without obesity and/or a non-surgical control precludes attributing changes in biomarkers solely to SG. Medication exposure during follow-up was not systematically recorded, and residual confounding from time-varying therapies (e.g., lipid-lowering, antiplatelet, antihypertensive, and glucose-lowering agents) cannot be excluded. The 12-month follow-up limits inference regarding the durability of vascular and biomarker changes, and unmeasured lifestyle modifications may also have contributed. Finally, several estimates, particularly the ROC analyses, showed wide confidence intervals, likely reflecting sample variability.

Future research should validate these findings in larger, multiethnic cohorts that include appropriate control arms and follow-up beyond one year. Studies should prospectively capture cardiometabolic medication exposure (initiation, discontinuation, and dose changes) to enable adjustment for time-varying pharmacotherapy and to test whether ox-LDL and PAI-1 provide incremental prognostic value beyond established risk factors and treatments. Exploration of longitudinal changes in PAI-1 and ox-LDL in relation to hard cardiovascular outcomes, such as imaging-confirmed atherosclerotic progression and major adverse cardiovascular events, would further define their potential clinical role. Integrating additional fibrinolytic markers (e.g., TAFI, D-dimer) and mechanistic studies of the adipose–vascular interface may deepen pathophysiological understanding and refine post-MBS risk stratification.

## Conclusion

In patients with obesity undergoing SG, significant improvements in vascular, inflammatory, and fibrinolytic markers were noted over one year. Reduced CIMT correlated with lower PAI-1 and oxidized LDL levels, suggesting reversibility of obesity-associated vascular risks. While ox-LDL effectively identified subclinical atherosclerosis, PAI-1 showed weaker performance and should not be used as an independent predictor. Instead, it may serve as a marker of postoperative metabolic improvement. Although these findings highlight the relationship between fibrinolysis, oxidative stress, and vascular remodeling following MBS, the clinical use of PAI-1 and ox-LDL as diagnostic biomarkers is still preliminary and requires further validation before being integrated into cardiovascular risk prediction models.

## Data Availability

The datasets generated and/or analyzed during the current study are available from the corresponding author upon reasonable request.
